# The Impact of Primary Treatment on Post-Traumatic Endophthalmitis in Children with Open Globe Injuries: A Study in China

**DOI:** 10.3390/ijerph16162956

**Published:** 2019-08-16

**Authors:** Lingling Zheng, Junlian Tan, Rongjiao Liu, Xueru Yang, Huiling He, Huiming Xiao, Liwen He

**Affiliations:** 1 Department of Ocular Trauma, State Key Laboratory of Ophthalmology, Zhongshan Ophthalmic Center, Sun Yat-sen University, 54# Xianlie South Road, Yuexiu District, Guangzhou 510060, China; 2 Department of Nursing, State Key Laboratory of Ophthalmology, Zhongshan Ophthalmic Center, Sun Yat-sen University, 7# Jinsui Road, Tianhe District, Guangzhou 510000, China

**Keywords:** pediatric open ocular injury, post-traumatic endophthalmitis, left-behind children

## Abstract

Post-traumatic endophthalmitis (PTE) is considered as one of the most serious complications after open globe injuries (OGIs), especially in children. Poor prognosis of this disease can lead to a variety of socioeconomic problems. This study aimed to investigate the clinical characteristics of pediatric OGIs and the factors associated with the development of PTE in China. 131 patients under 14 years old and needed hospitalization for management of OGIs were enrolled. There were 90 males and 41 females. 44 patients were left-behind children, the majority were 3–6 years old (*n* = 71, 71/131, 54.2%) and living in rural area (*n* = 106, 106/131, 80.9%). After injury, 82 patients received primary repair within 24 h, with the remaining 49 patients receiving primary repair after 24 h. Eventually, there were 28 (28/131, 21.4%) patients presented with PTE. In those 49 patients, the frequency of PTE achieving 32.7% (16/49). Univariate analysis showed that the timing of primary repair is significantly associated with the development of PTE (*p* < 0.05). Moreover, left-behind children took higher risks in having delayed treatment that over 24 h after OGIs (OR = 2.466, 95% CI: 1.16–5.26). Reducing the time before primary repair is a useful strategy to prevent the development of PTE. Special supervision is needed for pre-school-aged boys living in rural areas, especially for left-behind children.

## 1. Introduction

Open globe injuries (OGIs) refer to injuries with at least one full thickness wound of the globe, which are considered the most common cause of preventable monocular blindness [1]. The incidence of OGIs in children has been reported to be 3.8/100,000 in the United States [2], accounting for 76%–83.75% of all ocular injuries [3,4]. The poor prognosis of OGIs in children also leads to a variety of socioeconomic problems, and this may be devastating in children. Psychosocial development may be affected in many children that suffer from visual impairments [5]. 

Post-traumatic endophthalmitis (PTE) is considered as one of the most serious complications after OGIs, and the incidence of this disease varies from 3.6%–54.16% in different studies [6,7]. Although advanced antibiotics and treatment techniques currently exist, children diagnosed with PTE were generally reported to have a poor outcome [8]. Some risk factors were reported to be associated with the development of PTE, such as the delayed timing of primary repair, a large wound size, a retained intraocular foreign body, the location of the wound, a rural location, the female gender, and an age greater than 50 years old [9].

However, only few studies have reported on pediatric OGIs in China, especially PTE in pediatric patients. In this study, we aimed to identify the characteristics of pediatric OGIs and the risk factors of PTE in pediatric groups in China.

## 2. Materials and Methods 

### 2.1. Study Design

This was a prospective study of pediatric OGIs in the department of ocular trauma of a tertiary ophthalmic hospital in China. From January to October 2017, there were 131 patients that met the necessary criteria and were enrolled in this study. Patients were eligible for this study if they were under 14 years old and needed hospitalization for the management of OGIs. We excluded those who were diagnosed with other eye diseases and whose caregivers refused to participate.

Data, including age, gender, location of residence, left-behind children status, location when injured, timing of primary repair, causative objects, diagnoses, initial visual acuity, and complications were collected from the clinical records of patients. Ocular abnormal status and findings were recorded from hospitalization through 3 months of the follow-up time after discharge.

### 2.2. Data Analysis

All statistical analyses were performed with SPSS software version 22.0 (Microsoft Corporation, New York, NY, USA). Data were presented in terms of the mean, frequency, percentage, and standard deviation. Categorical variables in this study were analyzed with the chi-square test, fisher’s exact test, and logistic regression model at the 95% confidence interval. A *p*-value of less than 0.05 was considered statistically significant.

### 2.3. Ethical Considerations

This study was approved by the ethics committee of the Zhongshan Ophthalmic Center of Sun Yat-sen University and conducted in accordance with the Declaration of Helsinki (Code of Ethics: 2019KYPJ050). Informed consent was obtained from the caregivers of the enrolled patients, and a thorough explanation of the study design and aims were provided.

## 3. Results

Table 1 demonstrates the clinical features of all the 131 patients examined, which included 90 males and 41 females (sex ratio (male/female), 2.20:1). There were 44 (44/131, 33.6%) left-behind children, majority of the patients (*n* = 71, 71/131, 54.2%) were 3–6 years old when diagnosed and living in a rural area (*n* = 106, 106/131, 80.9%). The sharp objects were the most predominant causative objects (*n* = 78, 78/131, 59.5%). With regard to the injury type, 105, 17, 4, and 5 patients were diagnosed as penetrating, rupture, perforating, and intraocular foreign body injuries, respectively. 

After injury, 82 patients received primary repair within 24 h, while remaining 49 patients received primary repair after 24 h. Eventually, there were 28 (28/131, 21.4%) patients presented with PTE. Out of those who received prompt treatment within 24 h, only 12 (12/82, 14.6%) were diagnosed with endophthalmitis, whereas in those longer than 24 h, the frequency of PTE achieving 32.7% (16/49).

Among 131 pediatric OGIs patients, univariate analysis showed that there was a significant relationship between the timing of primary repair and the development of PTE in pediatric OGIs patients, while other variables did not indicate a significant difference between groups (Table 1 and Table 2). A longer time before primary repair (longer than 24 h) was significantly associated with the development of PTE (*p* < 0.05). Compared to the patients that received primary repair within 24 h, the risk of development of PTE in those who received primary repair after 24 h increased 1.828 times (OR = 2.828, 95% CI: 1.2–6.7) (Table 2).

Moreover, we analyzed the relative factors of the timing of primary repair (Table 3). The demographic information and injury features were investigated with regard to their relation to the timing of primary repair. There was a significant difference between the two groups in terms of left-behind children status (*p* < 0.05). As listed in Table 3, the results from the logistic regression analysis showed that left-behind children were more likely than others to have a delay in primary repair after injury (OR = 2.466, 95% CI: 1.16–5.26).

## 4. Discussion

OGIs account for more than 80% of ocular trauma, continuing to cause severe permanent visual loss [10]. It is well known that ocular trauma commonly arises in preschool male children, consistent with previous studies [11]. Our study demonstrated that 68.7% of OGI patients were boys, and 54.2% of OGIs occurred in children aged 3–6 years old. Pre-school-aged boys are full of curiosity about the world but lacking awareness of security, leading them to be more vulnerable to unintentional injuries than other children [12,13]. Moreover, the prevalence of OGIs commonly possessed obvious area differences. The majority of ocular trauma cases occurred in rural areas [14,15]. Lack of an awareness of security and playing outside without necessary supervision contribute to OGIs incidence. 

OGIs were detected most frequently in children injured by sharp objects, such as scissors, knives, and crushed glasses. Penetrating wounds were the most common type of open globe injuries in most previous relevant studies [16]. In our study, the overall incidence of penetrating wounds was 80.1%, followed by rupture (13.0%), which was similar to another study [17]. It was difficult to properly assess the initial visual acuity (VA) of children at present, and only 61.8% of OGIs children had a record of VA. This may be related to the lack of cooperation from these children because of fear and pain after OGIs [18]. The VA of most patients in our study was less than 1/200, which was in agreement with the findings of other studies [19,20].

Endophtalmitis is a potential blinding complication characterized by the inflammation, colonization and irreversible destruction by an infectious agent, which is uncommon, but one of the most severe complications after OGIs [21]. In our study, the incidence of PTE after OGIs was 21.4% compared to 3.6% to 54.16% reported by other studies from different institutions [6,7]. Bhagat et al. found that the timing of primary repair less than 24 h was considered one of the protective factors for the development of PTE [22]. In our study, we also found a statistically significant relationship between the timing of the primary repair and the development of PTE. For those who received prompt treatment within 24 h, only 12 (12/82, 14.6%) were diagnosed with endophthalmitis, whereas for those who received treatment longer than 24 h, the frequency of PTE reached 32.7% (16/49).

Furthermore, we also investigated the factors associated with the timing of primary repair. In this study, a significant relationship was found between the timing of primary repair and left-behind children status. Left-behind children are a social problem in China, especially in rural China [23]. Since the early 1990s, with rapid economic development and social changes in China, a large number of adults have moved into cities or other locations for better-paying jobs. An increasing number of left-behind children appeared as a result of this phenomenon [24]. The number of left-behind children who live without one or both parents has exploded to 60 million in China [25]. 

In our study, 33.6% of patients were left-behind children, which may be explained by the lack of supervision. Children who were not living with their parents had a higher risk of delaying primary repair more than 24 h after OGIs, which might result in severe consequences. Left-behind children are more vulnerable to unintentional injuries than other children, including ocular trauma [24]. According to a study of non-fatal injuries, left-behind children had a higher rate of injuries than children living with both parents in China [26]. Parents play an important role in injury prevention by not only providing safety education and supervision but also ensuring timely primary treatment when injuries occur. The health-seeking behavior among left-behind children is poor in China [25]. Therefore, the risk for serious complications including the development of PTE increased with the timing of primary repair delayed after eye injuries.

This study has several potential limitations related to the prospective study design. We collected data from the first hospitalization through 3 months of follow-up time after discharge. It was difficult to obtain additional follow-up data beyond 3 months. Furthermore, the results of this study should not be interpreted to represent the entirety of the pediatric OGIs in China because the number of subjects for this study was small. In addition, we did not consider the data from any cases that were treated in other hospitals.

## 5. Conclusions

After accounting for a number of cases of blindness or vision loss in children, OGIs can be avoided, and PTE is preventable after injuries. Special care and supervision are needed for pre-school-aged boys living in rural areas, especially for left-behind children. Reducing the time before primary repair is a useful strategy to prevent the development of PTE.

## Figures and Tables

**Table 1:** Clinical features of pediatric open globe injuries patients (*n* = 131).

Variable	$\bar{x}\pm s$	*n* (%)	PTE (*n* (%))		
			With (*n* = 28)	Without (*n* = 103)	*p*-Value *
Gender					1.000
Male		90 (68.7%)	19 (67.9%)	71 (68.9%)	
Female		41 (31.3%)	9 (32.1%)	32 (31.1%)	
Age (years)	5.51 ± 2.99				0.520
1–2		18 (13.7%)	4 (14.3%)	14 (13.6%)	
3–6		71 (54.2%)	16 (57.1%)	55 (53.4%)	
7–10		34 (26.0%)	8 (28.6%)	26 (25.2%)	
11–13		8 (6.1%)	0 (0.0%)	8 (7.8%)	
Left-behind children					0.242
Yes		44 (33.6%)	12 (42.9%)	32 (31.1%)	
No		87 (66.4%)	16 (57.1%)	71 (68.9%)	
Location of residence					0.141
Urban		25 (19.1%)	3 (10.7%)	22 (21.4%)	
Rural		106 (80.9%)	25 (89.3%)	81 (78.6%)	
Location when injured					0.625
Home		77 (58.8%)	17 (60.7%)	60 (58.3%)	
Outdoors		33 (25.2%)	7 (25.0%)	26 (25.2%)	
School		15 (11.4%)	4 (14.3%)	11 (10.7%)	
Undetermined		6 (4.6%)	0 (0.0%)	6 (5.8%)	
Causative objects					0.886
Sharp objects		78 (59.5%)	16 (57.1%)	62 (60.2%)	
Blunt objects		14 (10.7%)	4 (14.3%)	10 (9.7%)	
Plant		16 (12.2%)	4 (14.3%)	12 (11.7%)	
Firework		4 (3.1%)	1 (3.6%)	3 (2.9%)	
Animals		4 (3.1%)	0 (0.0%)	4 (3.9%)	
Undetermined		15 (11.4%)	3 (10.7%)	12 (11.6%)	
Timing of primary repair					0.014
≤24 h		82 (63.4%)	12 (42.9%)	70 (68.0%)	
>24 h		49 (36.6%)	16 (57.1%)	33 (22.0%)	
Injury type					0.650
Penetrating		105 (80.1%)	25 (89.3%)	80 (77.6%)	
Rupture		17 (13.0%)	2 (7.1%)	15 (14.6%)	
Perforating		4 (3.1%)	0 (0.0%)	4 (3.9%)	
Intraocular foreign body		5 (3.8%)	1 (3.6%)	4 (3.9%)	
Visual acuity					0.782
>20/40		1 (0.8%)	0 (0.0%)	1 (1.0%)	
20/200–20/50		6 (4.6%)	1 (3.6%)	5 (4.9%)	
1/200–19/200		7 (5.3%)	1 (3.6%)	6 (5.8%)	
Light perception–Finger count		66 (50.3%)	12 (42.8%)	54 (52.4%)	
No light perception		1 (0.8%)	0 (0.0%)	1 (1.0%)	
Undetermined		50 (38.2%)	14 (50.0%)	36 (34.9%)	

* Chi-square test for categorical variables. Fisher’s exact test was used when necessary. PTE = Post-traumatic endophthalmitis.

**Table 2:** Logistic regression analysis of post-traumatic endophthalmitis.

Variable	PTE		β	SE ^1^	OR (95% CI) ^2^	*p*-Value
	With	Without				
Timing of primary repair						0.017
≤24 h	12	70	1 (Ref.) ^3^	1 (Ref.)	1 (Ref.)	
>24 h	16	33	1.040	0.436	2.828(1.202–6.652)	

^1^ SE = Standard Error. ^2^ OR (95% CI) = Odds Ratio (95% Confidence Interval). ^3^ Ref. = Reference.

**Table 3:** Logistic regression analysis of the timing of primary repair.

Variable	Timing of Primary Repair		β	SE ^1^	OR (95% CI) ^2^	*p*-Value
	≤24 h	>24 h				
Left-behind						
Yes	21	23	1 (Ref.) ^3^	1 (Ref.)	1 (Ref.)	0.019 *
No	61	26	0.903	0.386	2.466(1.157–5.258)	
Gender						0.797
Male	56	34	1 (Ref.)	1 (Ref.)	1 (Ref.)	
Female	26	15	−0.100	0.403	0.905(0.411–1.993)	
Age(years)						0.962
1–6	55	34	1 (Ref.)	1 (Ref.)	1 (Ref.)	
7–14	27	15	−0.019	0.402	0.981(0.446–2.157)	
Location of residence						0.182
Urban	19	6	1 (Ref.)	1 (Ref.)	1 (Ref.)	
Rural	63	43	0.694	0.519	2.001(0.723–5.538)	

^1^ SE = Standard Error. ^2^ OR (95% CI) = Odds Ratio (95% Confidence Interval). ^3^ Ref. = Reference. * *p* < 0.05.
